# Death of a Fœtus from Smallpox

**Published:** 1856-05

**Authors:** 


*Death of a Fœtus from Smallpox.*—M. Blot relates the following example of this to the *Societe de Biologie*:

The mother of the little patient, previously in good health, and six months advanced in her second pregnancy, was attacked with smallpox, on the 17th of July, 1854. She hadnot been vaccinated, but the attack was not severe, and she recovered without any secondary fever. During her illness, the movements of the child were more continuous than they were before, but not so energetic; during her convalescence, the same movements became more and more feeble, until they ceased altogether, and the child was felt to fall towards the side on which the mother happened to lay. Two days after this cessation labor came on unexpectedly, and the mother removed to the "Clinique d'Accouchements," where she was presently delivered of a male fœtus of the six and a half or seventh month, *covered with pustules of smallpox.* This fœtus presented unequivocal evidence of having been recently alive, and there was every reason to believe that it had had smallpox at the same time as its mother, and that it had died from this cause.

[*Gazette Med. de Paris.*

